# Multifaceted regulatory functions of CsBPC2 in cucumber under salt stress conditions

**DOI:** 10.1093/hr/uhad051

**Published:** 2023-03-15

**Authors:** Shuzhen Li, Mintao Sun, Li Miao, Qinghua Di, Lijun Lv, Xianchang Yu, Yan Yan, Chaoxing He, Jun Wang, Aokun Shi, Yansu Li

**Affiliations:** State Key Laboratory of Vegetable Biobreeding, Institute of Vegetables and Flowers, Chinese Academy of Agricultural Sciences, Beijing 100081, China; Ganzhou Key Laboratory of Greenhouse Vegetable, College of Life Science, Gannan Normal University, Ganzhou 341000, China; State Key Laboratory of Vegetable Biobreeding, Institute of Vegetables and Flowers, Chinese Academy of Agricultural Sciences, Beijing 100081, China; Collaborative Innovation Center for Efficient and Green Production of Agriculture in Mountainous Areas of Zhejiang Province, College of Horticulture Science, Zhejiang Agriculture and Forestry University, Hangzhou 311300, Zhejiang, China; State Key Laboratory of Vegetable Biobreeding, Institute of Vegetables and Flowers, Chinese Academy of Agricultural Sciences, Beijing 100081, China; State Key Laboratory of Vegetable Biobreeding, Institute of Vegetables and Flowers, Chinese Academy of Agricultural Sciences, Beijing 100081, China; State Key Laboratory of Vegetable Biobreeding, Institute of Vegetables and Flowers, Chinese Academy of Agricultural Sciences, Beijing 100081, China; State Key Laboratory of Vegetable Biobreeding, Institute of Vegetables and Flowers, Chinese Academy of Agricultural Sciences, Beijing 100081, China; State Key Laboratory of Vegetable Biobreeding, Institute of Vegetables and Flowers, Chinese Academy of Agricultural Sciences, Beijing 100081, China; State Key Laboratory of Vegetable Biobreeding, Institute of Vegetables and Flowers, Chinese Academy of Agricultural Sciences, Beijing 100081, China; State Key Laboratory of Vegetable Biobreeding, Institute of Vegetables and Flowers, Chinese Academy of Agricultural Sciences, Beijing 100081, China; State Key Laboratory of Vegetable Biobreeding, Institute of Vegetables and Flowers, Chinese Academy of Agricultural Sciences, Beijing 100081, China

## Abstract

BASIC PENTACYSTEINE (BPC) transcription factors are essential regulators of plant growth and development. However, BPC functions and the related molecular mechanisms during cucumber (*Cucumis sativus* L.) responses to abiotic stresses, especially salt stress, remain unknown. We previously determined that salt stress induces *CsBPC* expression in cucumber. In this study, *Csbpc2* transgene-free cucumber plants were created using a CRISPR/Cas9-mediated editing system to explore CsBPC functions associated with the salt stress response. The *Csbpc2* mutants had a hypersensitive phenotype, with increased leaf chlorosis, decreased biomass, and increased malondialdehyde and electrolytic leakage levels under salt stress conditions. Additionally, a mutated *CsBPC2* resulted in decreased proline and soluble sugar contents and antioxidant enzyme activities, which led to the accumulation of hydrogen peroxide and superoxide radicals. Furthermore, the mutation to *CsBPC2* inhibited salinity-induced PM-H^+^-ATPase and V-H^+^-ATPase activities, resulting in decreased Na^+^ efflux and increased K^+^ efflux. These findings suggest that CsBPC2 may mediate plant salt stress resistance through its effects on osmoregulation, reactive oxygen species scavenging, and ion homeostasis-related regulatory pathways. However, CsBPC2 also affected ABA signaling. The mutation to *CsBPC2* adversely affected salt-induced ABA biosynthesis and the expression of ABA signaling-related genes. Our results indicate that CsBPC2 may enhance the cucumber response to salt stress. It may also function as an important regulator of ABA biosynthesis and signal transduction. These findings will enrich our understanding of the biological functions of BPCs, especially their roles in abiotic stress responses, thereby providing the theoretical basis for improving crop salt tolerance.

## Introduction

Soil salinity is an environmental factor with extremely adverse effects on agricultural production worldwide [1]. Excessive salinity can cause osmotic stress, ion toxicity, and oxidative damage to plants, which seriously hinders growth, development, and crop productivity [2–4]. However, plants have evolved many physiological and molecular mechanisms that protect against detrimental environmental conditions. When plants suffer from salt stress, many signaling pathways and genes are activated to modulate ion homeostasis through the salt overly sensitive (SOS)-mediated pathway, increase the production of osmoprotectants (e.g. proline) that function as osmotic regulators, enhance antioxidant enzyme activities, increase the reactive oxygen species (ROS) scavenging capacity, and induce stomatal closure mediated by the abscisic acid (ABA) biosynthesis and signaling pathway [5–7]. Although there has recently been substantial progress in the research conducted to clarify plant salt stress responses, the utility of the identified and functionally characterized stress-related genes for increasing salt tolerance depends on genetic engineering techniques.

BASIC PENTACYSTEINE (BPC)/BARLEY B RECOMBINANT (BBR) is a family of plant-specific transcription factors (TFs) that bind to promoters comprising GA-rich sequences [8]. The BPC TF genes form a small gene family in many species. In *Arabidopsis thaliana*, the seven BPC family members that are divided into the following three classes according to their C-terminal amino acid sequence: class I (BPC1/2/3), class II (BPC4/5/6), and class III (only BPC7) [9]. The seven BPC TFs have a highly conserved C-terminal BPC domain that is required for DNA binding [10]. This domain includes five cysteine residues that recognize the GAGA motif [11]. In contrast, the N-terminal domains of BPC TFs vary considerably.

Several studies have demonstrated that BPC TFs are vital regulators of plant growth and developmental processes mainly affecting seed size and dormancy, inflorescence meristem and floral organ development, flowering time, lateral root formation, leaf morphology, plant architecture, and fertility [9, 12–17]. Mutations to *BPC* genes cause both vegetative and reproductive defects [14]. Many BPC TF target genes important for development have been identified, such as the ovule identity gene *SEEDSTICK* (*STK*) and *INNER NO OUTER* (*INO*) [8, 9, 18, 19], the seed development-related gene *LEAFY COTYLEDON 2* (*LEC2*) [20], the floral organ development gene *BREVIPEDICELLUS*/*KNAT1* (*BP*) and *SHOOTMERISTEMLESS* (*STM*) [17], and the flowering-related gene *OsLFL1* [12]. Moreover, BPCs also control plant responses to hormones, including ethylene [14], cytokinins [17, 21], and brassinosteroids [11]. Although the numerous roles of BPCs in developmental processes have been identified, functions of BPCs in abiotic stress responses are rarely studied.

We previously reported that the expression levels of all cucumber *BPC* (*CsBPC*) genes increase following an exposure to various abiotic stresses, suggesting that CsBPCs mediate plant abiotic stress responses [22]. A recent study indicated that BPC1/BPC2 positively regulate *A. thaliana* salinity resistance with suppressing *GALACTAN SYNTHASE 1* (*GALS1*) expression [23], but another study showed that BPC2 negatively regulates osmotic stress resistance with suppressing *LEA4–5* expression [24]. These results reflect the importance of BPCs for plant stress resistance. However, BPC functions during stress responses and the underlying regulatory mechanisms must be further explored. In this study, *Csbpc2* transgene-free cucumber plants were produced via CRISPR/Cas9-mediated editing. We subsequently analysed osmotic adjustments, ROS scavenging, ion homeostasis regulation, and the ABA signaling pathway to further elucidate CsBPC2 functions during salt stress responses. Our results showed that a mutation to *CsBPC2* adversely affects osmotic adjustments, ROS scavenging, and ion homeostasis, while also inhibiting salt-induced ABA biosynthesis and the transcription of ABA signaling-related genes in cucumber seedlings. Considered together, these findings imply that CsBPC2 positively regulates the salt stress response, possibly through its effects on the ABA signaling pathway.

## Results

### Creation of *Csbpc2* homozygous mutants via CRISPR-Cas9

**Figure 1:**
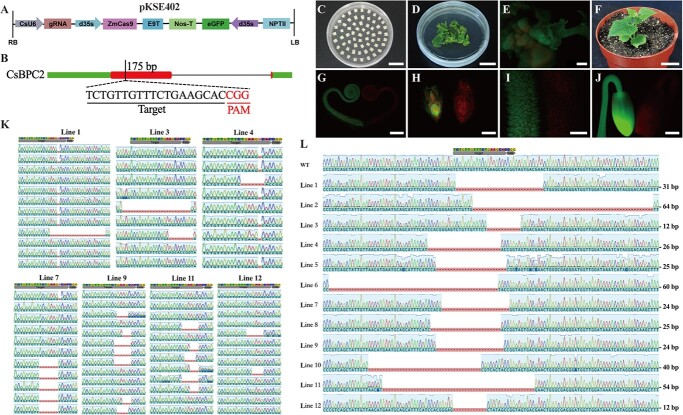
Creation of *Csbpc2* transgene-free cucumber plants using the CRISPR/Cas9 system. **A** Schematic of the CRISPR/Cas9 binary vector pKSE402. The vector was modified by inserting a GFP cassette, which was used to screen for transgenic plants. **B***CsBPC2* gene structure and target site. Black lines and red boxes represent introns and exons, respectively. The 5′- and 3′-untranslated regions are indicated by green boxes. The target site (vertical black bar) is located at 175 bp in the first exon of *CsBPC2*. The PAM and target sequence is provided in red and black letters, respectively. **C**–**F***Agrobacterium tumefaciens*-mediated cucumber transformation. **C** Preparation of explants, scale bar = 2 cm; **D** 16-day-old explants cultured on shoot regeneration medium, scale bar = 2 cm; **E** GFP fluorescence of regenerated shoots, scale bar = 0.2 mm; **F** Transgenic plant, scale bar = 2 cm. **G**–**J** Identification of GFP-positive plants, scale bar = 0.5 mm. The GFP fluorescence in the tendrils (**G**), male flowers (**H**), ovaries (**I**), and germinated seeds (**J**) of T_0_ plants was detected using the Leica MZ10F stereomicroscope. Plants with green fluorescence (left side) were transgenic lines, whereas plants with red fluorescence (right side) were wild-type lines. **K** Edited *CsBPC2* target site in T_0_ plants. The wild-type sequence (i.e. control) is presented at the top, whereas the remaining sequences were from individual clones. Sequences were aligned using the Geneious software. The target and PAM sequences are indicated above the sequence chromatograms. Red dashes represent inserted or deleted nucleotides. **L** Identification of *Csbpc2* transgene-free homozygous mutants on the basis of Sanger sequencing. WT, wild-type; Lines 1–12, transgene-free homozygous mutants. Red dashes represent deleted nucleotides (the number of deleted nucleotides is indicated on the right).

Previous research showed that *CsBPC2* is the most highly expressed *CsBPC* gene following an exposure to common abiotic stresses and hormones [22]. The CRISPR/Cas9-*CsBPC2* vector with one single-guide RNA (sgRNA) cassette was constructed (Fig. 1A and B) and transferred into cucumber plants (Fig. 1C–F) to functionally characterize *CsBPC2*, especially in response to salt stress. Because a GFP cassette was inserted into the pKSE402 vector (Fig. 1A), GFP fluorescence was examined when explants were co-cultured for 16 days (Fig. 1D and E). Then, fluorescent buds were detached from explants and cultured further. Finally, we obtained 12 independent GFP-positive cucumber plants from approximately 12 800 seeds (Fig. 1G–J). The transformation efficiency (about 1‰) was similar to that obtained by Hu *et al.* [25]. The sequencing analysis detected mutations in seven lines (Fig. 1K), which were selfed. The GFP fluorescence in their seeds indicated that the segregation ratio of fluorescent and non-fluorescent seeds was approximately 3:1. The T_1_ progeny seedlings were validated by PCR and Sanger sequencing. The results showed that mutations occurred in both fluorescent and non-fluorescent seedlings. Additionally, new mutations were also detected in both seedling types. As in Hu’s study [25], the co-segregation of Cas9 and GFP was used to screen for transgene-free mutants. Accordingly, we selected non-fluorescent mutated plants for self-pollinations, which ultimately resulted in transgene-free *Csbpc2* homozygous mutants with large deleted fragments (Fig. 1L).

### CsBPC2 positively regulates salt tolerance in cucumber

**Figure 2:**
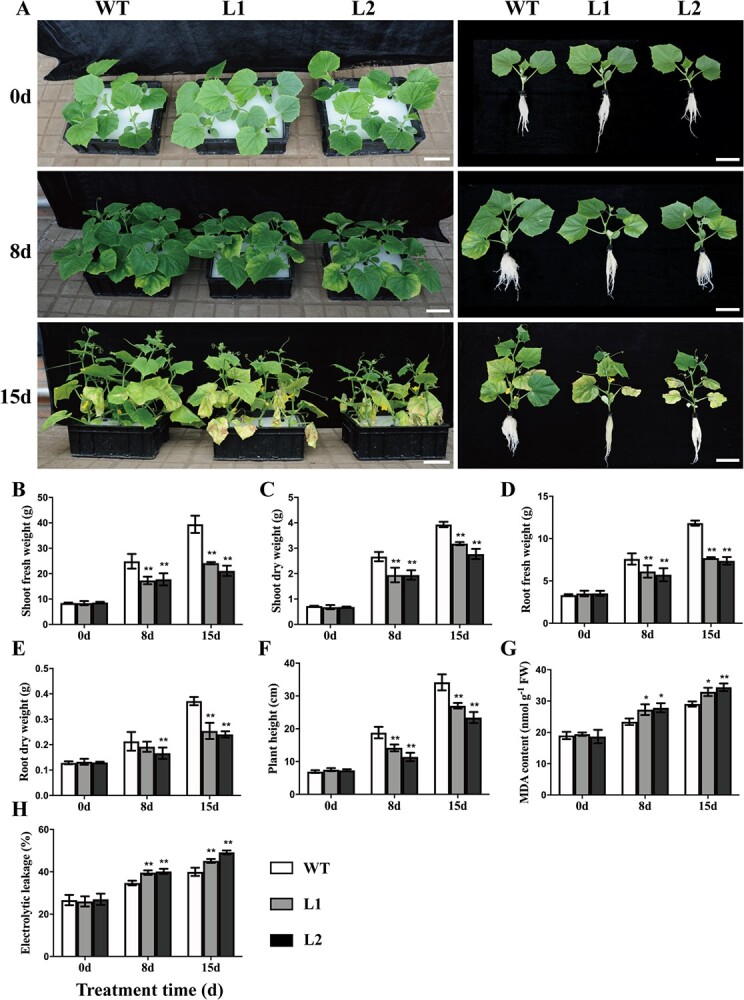
The mutation to *CsBPC2* decreased cucumber salt stress tolerance. **A** Phenotypes of WT and *Csbpc2* mutant plants under saline conditions, scale bar = 10 cm. Cucumber seedlings were treated with 100 mM NaCl and then photographed before and after 8- and 15-day treatments. WT, wild-type; L1 and L2, *Csbpc2* mutant Line 1 and Line 2 (corresponding to the information provided in Fig. 1L and supplement data S1), with 31 and 64 deleted nucleotides, respectively. **B**–**C** Cucumber shoot fresh and dry weights. **D**–**E** Cucumber root fresh and dry weights. **F** Plant height. **G** Malondialdehyde (MDA) concentrations in the leaves. **H** Electrolytic leakage (EL) in the leaves. Values are presented as the mean ± SD (*n* = 3). * and **, significant at *P* < 0.05 and *P* < 0.01 (compared with the WT control), respectively.

To explore whether CsBPC2 contributes to plant responses to salt stress, 12 transgene-free homozygous *Csbpc2* mutants were generated using the CRISPR/Cas9 system (Fig. 1L). We selected Line 1 (L1) and Line 2 (L2) for the subsequent experiments because they contained a frameshift mutation and a large deleted fragment (sequences of nucleic acid and protein are listed in Data S1, see online supplementary). Under growth conditions, there were no obvious phenotypic differences between wild-type (WT) and mutant plants (Fig. 2A). However, the 8-day treatment with 100 mM NaCl inhibited the growth of L1 and L2, as demonstrated by the extensive leaf chlorosis, decreased leaf area, and decrease in the number of leaves (compared with the WT control) (Fig. 2A). Similarly, the plant height and biomass were significantly lower for L1 and L2 than for the WT control (Fig. 2B–F). The inhibitory effects on the growth of the WT, L1, and L2 plants increased as the duration of the stress treatment increased. More specifically, on day 15, almost all of the older basal leaves of the L1 and L2 plants were yellowing and dying or had fallen off (Fig. 2A). The 15-day salt treatment resulted in increases in the following L1, L2, and WT seedling traits: plant height (258.8%, 219.5%, and 392.8%, respectively) (Fig. 2F), shoot fresh weight (187.6%, 145.2%, and 366.5%, respectively) (Fig. 2B), shoot dry weight (366.6%, 303.7%, and 442.7%, respectively) (Fig. 2C), root fresh weight (119.1%, 108.8%, and 256.7%, respectively) (Fig. 2D), and root dry weight (91.3%, 85.2%, and 189.0%, respectively) (Fig. 2E). These results indicated the *Csbpc2* mutants were much more sensitive to salinity stress than WT control. The malondialdehyde (MDA) and electrolytic leakage (EL) levels have been used as indicators of membrane damage due to salt stress [26]. Under growth conditions, there were no significant differences in MDA content (Fig. 2G) and EL level (Fig. 2H) between the WT and mutant (L1 and L2) plants. In contrast, under salinity conditions, the MDA and EL levels increased in all plants, but the increases were significantly greater for L1 and L2 than for the WT control, implying that the mutation to *CsBPC2* destabilized membrane systems in salt-stressed cucumber seedlings.

### CsBPC2 affects osmoprotectant biosynthesis under salt stress conditions

**Figure 3:**
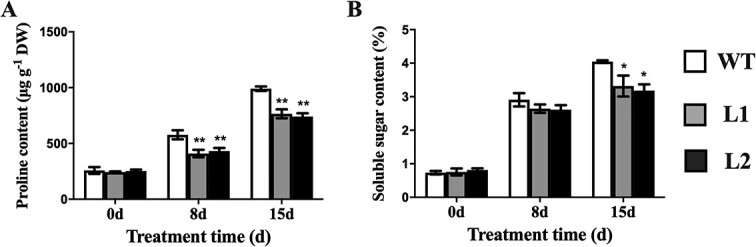
*CsBPC2* is important for osmoprotectant biosynthesis under salt stress conditions. The proline (**A**) and soluble sugar (**B**) contents in leaves of WT and *Csbpc2* mutant plants before and after 8- and 15-day salt treatments. Values are presented as the mean ± SD (n = 3). * and **, significant at *P* < 0.05 and *P* < 0.01 (compared with the WT control), respectively.

Under normal conditions, there were no apparent differences in the proline and soluble sugar contents between the WT control and *Csbpc2* mutants (Fig. 3). After the exposure to saline conditions, the proline and soluble sugar levels increased dramatically in all plants, with a significantly greater increase in the WT control than in the *Csbpc2* mutants. Specifically, the 8-day salt treatment increased the proline contents of the L1, L2, and WT plants by 67.9%, 70.6%, and 123.7%, respectively. There was no significant difference in the soluble sugar contents. After the 15-day salt treatment, the proline contents of the L1, L2, and WT plants increased by 214.2%, 192.3%, and 284.1%, respectively (compared with the corresponding contents on day 0). Furthermore, the soluble sugar contents of the L1, L2, and WT plants increased by 3.4-, 2.9-, and 4.5-fold, respectively.

These findings indicated that salt stress induced the accumulation of proline and soluble sugars, but a mutated *CsBPC2* inhibited the accumulation of these two substances.

### CsBPC2 positively regulates ROS detoxification under salt stress conditions

**Figure 4:**
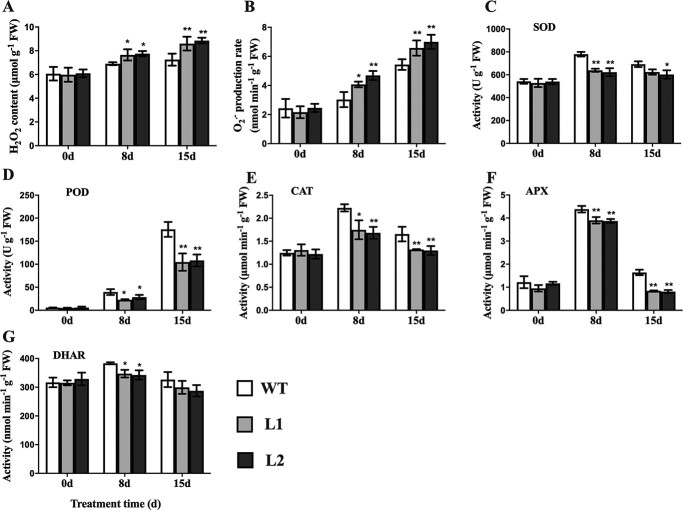
The mutation to *CsBPC2* decreased the ROS scavenging capacity of salt-stressed cucumber. **A**–**B** Hydrogen peroxide (H_2_O_2_) contents (**A**) and superoxide anion radical (O_2_^.−^) production rates (**B**) in leaves of WT and *Csbpc2* mutant plants exposed to salinity stress for 0, 8, and 15 days. **C**–**G** Superoxide dismutase (SOD; **C**), peroxidase (POD; **D**), catalase (CAT; **E**), ascorbate peroxidase (APX; **F**), and dehydroascorbate reductase (DHAR; **G**) activities in leaves of WT and *Csbpc2* mutant plants exposed to salinity stress for 0, 8, and 15 days. Values are presented as the mean ± SD (n = 3). * and **, significant at *P* < 0.05 and *P* < 0.01 (compared with the WT control), respectively.

Salinity stress induces the excessive accumulation of ROS, which leads to cellular damages. Under normal conditions, the H_2_O_2_ (Fig. 4A) and O_2_^.−^ (Fig. 4B) contents did not differ significantly between the WT control and the *Csbpc2* mutants. However, under salt stress conditions, the mutants accumulated more H_2_O_2_ and O_2_^.−^ than the WT plants. Because plant antioxidant enzyme systems can eliminate excessive ROS, we analysed the activities of the antioxidant enzymes superoxide dismutase (SOD) (Fig. 4C), peroxidase (POD) (Fig. 4D), catalase (CAT) (Fig. 4E), ascorbate peroxidase (APX) (Fig. 4F), and dehydroascorbate reductase (DHAR) (Fig. 4G) in the WT and *Csbpc2* mutant plants under normal and saline conditions. In the absence of salt stress, there were no significant differences in the activities of all analysed enzymes. Following the exposure to salt stress, the SOD, CAT, APX, and DHAR activities in the WT, L1, and L2 plants increased on day 8, but decreased on day 15. The POD activity increased substantially on days 8 and 15. However, at all time-points, the enzyme activities were higher in the WT control than in the L1 and L2 mutants. Accordingly, compared with the WT control, the *Csbpc2* mutants accumulated more ROS under saline conditions. Moreover, CsBPC2 modulates the scavenging of ROS in response to salt stress by regulating SOD, POD, CAT, APX, and DHAR activities.

### CsBPC2 regulates ion homeostasis under salt stress conditions

**Figure 5:**
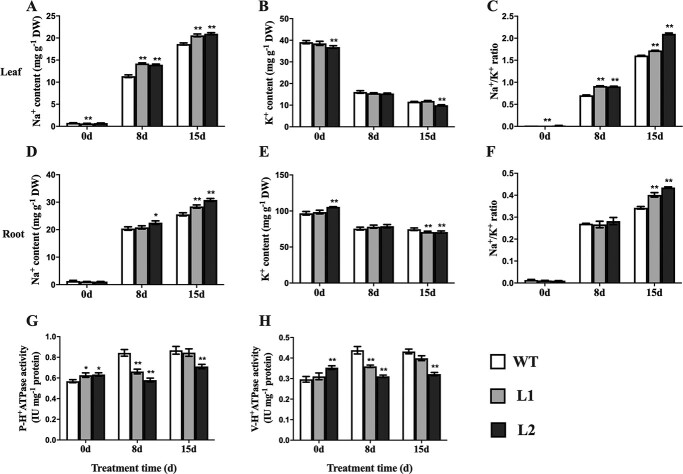
*CsBPC2* encodes a regulator of ion homeostasis under saline conditions. **A**–**C** Na^+^ (**A**) and K^+^ (**B**) contents and Na^+^/K^+^ ratios (**C**) in leaves of WT and *Csbpc2* mutant plants under salt stress conditions. **D**–**F** Na^+^ (**D**) and K^+^ (**E**) contents and Na^+^/K^+^ ratios (**F**) in roots of WT and *Csbpc2* mutant plants under salt stress conditions. **G**–**H** Proton pump activities in the plasma membrane (PM-H^+^-ATPase; **G**) and vacuolar membrane (V-H^+^-ATPase; **H**) in leaves of WT and *Csbpc2* mutants under salt stress conditions. Values are presented as the mean ± SD (*n* = 3). * and **, significant at *P* < 0.05 and *P* < 0.01 (compared with the WT control), respectively.

Salinity stress leads to the excessive accumulation of sodium ions (Na^+^) in leaves, which inhibits the absorption of beneficial potassium ions (K^+^), with the resulting ion toxicity negatively affecting plant growth. We examined the Na^+^ and K^+^ contents in the leaves and roots of WT and *Csbpc2* mutant plants exposed to salinity stress. Compared with the untreated samples, the Na^+^ contents increased considerably in all leaves and roots, which was in contrast to the substantial decrease in the K^+^ contents (Fig. 5). These changes resulted in a significant increase in the Na^+^/K^+^ ratio. However, compared with the WT control, the L1 and L2 mutants accumulated more Na^+^ (Fig. 5A and D) and less K^+^ (Fig. 5B and E) in the leaves and roots under saline conditions. Hence, the Na^+^/K^+^ ratio (Fig. 5C and F) was higher in the mutants than in the WT plants.

The maintenance of cytosolic Na^+^/K^+^ homeostasis is essential for plant salt tolerance. The exclusion or compartmentalization of Na^+^ in vacuoles can alleviate the toxic effects of Na^+^. Both PM-H^+^-ATPase and V-H^+^-ATPase generate a transmembrane proton electrochemical gradient, which can promote the transmembrane transport of Na^+^, thereby maintaining ion homeostasis. Thus, we examined the salt-induced PM-H^+^-ATPase (Fig. 5G) and V-H^+^-ATPase (Fig. 5H) activities in the leaves of WT and *Csbpc2* mutant plants. Under normal conditions, the PM-H^+^-ATPase and V-H^+^-ATPase activities were higher in L1 and L2 than in the WT control. However, under salt stress conditions, the PM-H^+^-ATPase and V-H^+^-ATPase activities were significantly higher in the WT control than in the L1 and L2 mutants. Compared with the enzyme activities in the WT plants on day 8 of the salt treatment, the PM-H^+^-ATPase activities were 27.3% and 45.5% lower in L1 and L2, respectively, whereas the V-H^+^-ATPase activities were 22.0% and 40.8% lower in L1 and L2, respectively. On day 15 of the salt treatment, the PM-H^+^-ATPase activities of L1 and L2 were respectively 2.6% and 22.1% lower than that of the WT control, while the V-H^+^-ATPase activities were respectively 8.4% and 34.2% lower than that of the WT control. These results suggest that CsBPC2 is important for maintaining ion homeostasis under salt stress conditions.

### CsBPC2 affects the net flux of Na^+^ and K^+^ under salt stress conditions

**Figure 6:**
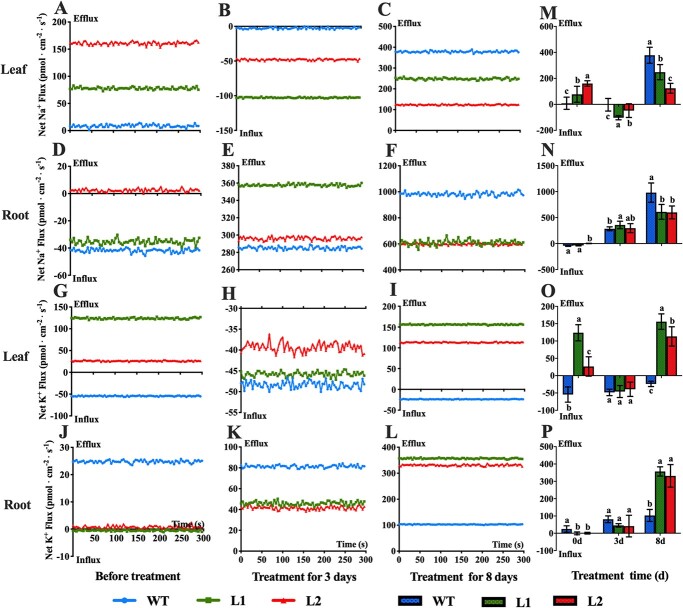
Effects of CsBPC2 on the net flux of Na^+^ and K^+^ in cucumber seedlings under salt stress conditions. **A**–**L** Net Na^+^ and K^+^ flux in the leaves and roots of WT and *Csbpc2* mutant plants under saline conditions. **M**–**P** Mean net Na^+^ and K^+^ flux in the samples presented in panels **A**–**L**. Values are presented as the mean ± SD (*n* = 8). Different letters indicate significant differences (*P* < 0.05).

The net flux of Na^+^ and K^+^ in the roots and leaves before and during the salt treatment was measured using non-invasive micro-test technology (NMT). The results showed that salt stress induced Na^+^ and K^+^ efflux in the WT and *Csbpc2* mutant roots. The Na^+^ and K^+^ efflux rates increased significantly as the duration of the salt stress treatment increased. After the 8-day salt treatment, the net Na^+^ and K^+^ efflux rates were, respectively, substantially lower and higher in the mutant roots than in the WT roots (Fig. 6D–F, J–L, N, and P). In the leaves under normal conditions, Na^+^ efflux was detected in the WT and *Csbpc2* mutant plants, but the Na^+^ efflux rate was much lower in the WT control than in the mutants. After a 3-day salt treatment, Na^+^ influx was observed in the WT and mutant plants, but the Na^+^ influx rate was considerably lower in the WT control than in the mutants. After 8 days of the salt stress treatment, Na^+^ efflux was detected in all plants, but the efflux rate was significantly higher in the WT control than in the mutants (Fig. 6A–C and M). In the leaves under normal conditions, K^+^ influx was observed in the WT plants, which was in contrast to the K^+^ efflux in the mutants. After 3 days of the salt stress treatment, there was no difference in the K^+^ influx detected in the WT and mutant plants. After an 8-day exposure to salt stress, K^+^ influx was observed in the WT plants, whereas K^+^ efflux was detected in the mutants (Fig. 6G–I and O). Thus, a mutated *CsBPC2* in cucumber apparently decreases the ability of plants to extrude Na^+^ and retain K^+^ under saline conditions.

### CsBPC2 affects ABA synthesis and ABA signaling-related gene expression under salt stress conditions

**Figure 7:**
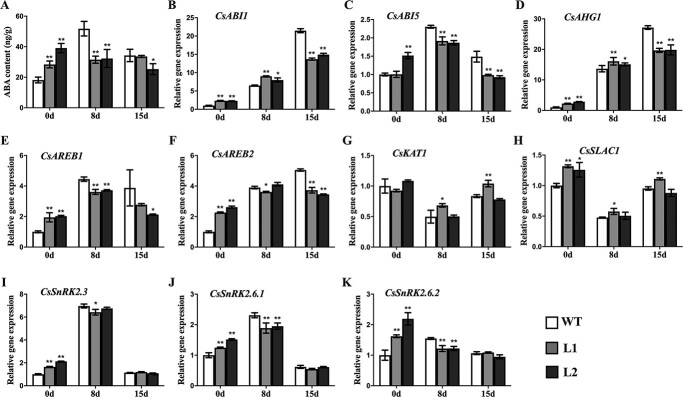
Effects of CsBPC2 on ABA contents and transcript levels of ABA signaling pathway-related genes under salt stress conditions. **A** ABA contents in leaves of WT and *Csbpc2* mutant plants after 0, 8, and 15 days of the salinity treatment. **B**–**K** Transcript levels of ABA signaling-related genes in the leaves of WT and *Csbpc2* mutant plants after 0, 8, and 15 days of the salinity treatment. The transcript levels in leaves of the non-stressed WT were set as 1. Values are presented as the mean ± SD (*n* = 3). * and **, significant at *P* < 0.05 and *P* < 0.01 (compared with the WT control), respectively.

To elucidate the involvement of CsBPC2 in ABA-mediated signal transduction under salinity conditions, the ABA contents and ABA signaling-related gene expression levels were analysed. The ABA contents were significantly higher in *Csbpc2* mutants than in WT plants before the salt treatment (Fig. 7A). However, under salt stress conditions, the ABA content in the WT control significantly increased on day 8 and then decreased, whereas there was only a minor change in the ABA content of the mutant plants. In addition, the ABA content was significantly higher in the WT control than in the mutant plants on day 8 of the salt treatment. These findings suggest that CsBPC2 helps regulate ABA synthesis under salt stress conditions, with mutations to *CsBPC2* leading to inhibited ABA synthesis. The qRT-PCR analysis revealed that *CsABI1* (Fig. 7B), *CsABI5* (Fig. 7C), *CsAHG1* (Fig. 7D), *CsAREB1* (Fig. 7E), *CsAREB2* (Fig. 7F), *CsSLAC1* (Fig. 7H), *CsSnRK2.3* (Fig. 7I), *CsSnRK2.6.1* (Fig. 7J), and *CsSnRK2.6.2* (Fig. 7K) were more highly expressed in the *Csbpc2* mutants than in the WT plants before the salt treatment, possibly because the high ABA concentration promoted the transduction of ABA signals. However, *CsABI1* (Fig. 7B), *CsABI5* (Fig. 7C), *CsAHG1* (Fig. 7D), *CsAREB1* (Fig. 7E), *CsAREB2* (Fig. 7F), *CsSnRK2.6.1* (Fig. 7J), and *CsSnRK2.6.2* (Fig. 7K) transcript levels were significantly lower in mutants than in WT plants following the salinity treatment (8 or 15 days), implying that CsBPC2 affects salt-induced signal transduction pathways.

## Discussion

### CsBPC2 is a positive regulator of cucumber salt tolerance

Crops are frequently exposed to diverse stresses that severely restrict productivity. However, plants have evolved several adaptive mechanisms that protect them from external stresses [27]. Many stress response-related genes have recently been identified and characterized, with implications for improving stress resistance of crops [1]. Although the multifaceted effects of the plant-specific BPC TFs on plant growth and development have been reported, the functions of these TFs in abiotic stress responses are unclear [23, 24]. We previously demonstrated that abiotic stresses (drought, salt, heat, and cold) induce *CsBPC* expression, implying BPC TFs help mediate abiotic stress responses [22], but the underlying mechanisms are unknown. Yan *et al.* [23] confirmed that in *A. thaliana*, BPC1/BPC2 increase salt tolerance by decreasing the accumulation of β-1,4-galactan, which negatively regulates salt resistance. Li *et al.* [24] showed that BPC2 decreases osmotic stress resistance with suppressing *LEA4–5* expression in *A. thaliana*. In the current study, which was conducted to clarify BPC functions during abiotic stress responses, *Csbpc2* transgene-free cucumber plants were created via CRISPR/Cas9-mediated editing (Fig. 1). There were no clear phenotypic differences between the WT and mutant plants under normal growth conditions, but under salt stress conditions, the *Csbpc2* mutants exhibited a hypersensitive phenotype (i.e. lower biomass and higher MDA and EL levels), indicating that CsBPC2 positively regulates salt stress tolerance (Fig. 2). Thus, BPC TFs might have complex regulatory effects on stress responses, which may vary among plant species and developmental stages. More studies are needed to explore BPC-mediated stress resistance. In the current study, we focused on osmoregulation, antioxidant regulation, ion homeostasis maintenance, and ABA signaling pathways to further elucidate BPC functions related to plant salt stress resistance.

### CsBPC2 affects osmoprotectant biosynthesis under salt stress

Osmotic adjustments are crucial for plant resistance to environmental stresses. Excessive salt ions reduce the osmotic potential of the soil, causing osmotic stress to plants. However, when plants are exposed to salt stress, many osmolytes will be synthesized. These osmolytes can effectively combine with free water, thus reducing the cell osmotic potential, enabling the cells to continue to absorb water from the outside, so as to maintain cell turgor and stabilize the structure of cells and proteins [28]. Proline levels often increase when plants suffer from various stresses, suggesting proline is an important osmoregulatory compound that alleviates the harmful effects of osmotic stress, thereby increasing plant stress tolerance [28–31]. Additionally, soluble sugars, which are the main products of photosynthesis in higher plants, are macromolecular substances required for plant growth and development, but they are also key osmoregulators that modulate the water potential and osmotic potential to enhance plant stress resistance [32, 33]. In this study, the levels of soluble sugar and proline in leaves of *Csbpc2* mutants and WT seedlings increased significantly as the salt stress treatment period increased, but both of these osmoregulatory substances were detected at significantly lower levels in the *Csbpc2* mutants than in the WT plants (Fig. 3). Accordingly, a mutated *CsBPC2* inhibits the synthesis of osmotic regulators induced by salt stress, which disrupts the cell osmotic potential. The subsequent inhibited uptake of water from the external hypertonic environment and water efflux lead to abnormal internal water levels. Therefore, *Csbpc2* mutants are more susceptible to salt stress than WT cucumber plants. Under the same salt stress condition, the *Csbpc2* mutant leaves had severe chlorotic lesions and were withered, dead, or had dropped from the plants.

### CsBPC2 helps in ROS scavenging under salt stress

Salinity stress also causes oxidative damage because it induces the accumulation of ROS (e.g. H_2_O_2_, O_2_^.−^, and OH^.−^), which leads to cell membrane structural damage and metabolic abnormalities that seriously affect plant growth and development [34–36]. Therefore, ROS scavengers are crucial for plant resistance to salt stress. Plants have evolved complex antioxidant systems comprising diverse enzymes, such as SOD, POD, CAT, APX, DHAR, MDHAR, and GR, that convert harmful ROS to water [37, 38]. In the present study, SOD, POD, CAT, APX, and DHAR activities were substantially lower in the *Csbpc2* mutants than in the WT plants under saline conditions, whereas the opposite trends were observed for the H_2_O_2_ and O_2_^.−^ levels (Fig. 4). These observations imply that CsBPC2 is vital for the detoxification of ROS. Moreover, mutations to *CsBPC2* adversely affect antioxidant pathways, resulting in decreased ROS scavenging. Thus, the decreased antioxidant enzyme activities and increased ROS contents in the *Csbpc2* mutants led to increased membrane lipid peroxidation and cell membrane damage, thereby increasing the susceptibility of the cucumber plants to salt stress.

### CsBPC2 maintains ion balance under salt stress

Plants under normal growth conditions have relatively low Na^+^ and high K^+^ levels. Maintaining an appropriate balance between these two ions is critical for plant growth and development. However, when plants are exposed to salt stress, excessive amounts of Na^+^ enter the root epidermal cells through ion channels, including the low-affinity K^+^ channel (AKT1), high-affinity K^+^ channel (HKT), and nonselective cation channel (NSCC). These ion channels simultaneously mediate the influx of Na^+^ and K^+^, which have a similar ionic radius. Therefore, the influx of large amounts of Na^+^ hinders the absorption of beneficial K^+^ ions, resulting in a decrease in the K^+^ content and a significant increase in the Na^+^/K^+^ ratio, which leads to ion toxicity [7]. Excessive Na^+^ accumulation or transport into shoots is prevented by restricting Na^+^ uptake, promoting Na^+^ efflux, and compartmentalizing Na^+^ in vacuoles [39]. The plasma membrane Na^+^/H^+^ antiporter SOS1 mediates Na^+^ efflux, which requires the proton gradient generated by the plasma membrane H^+^-ATPase (PM-H^+^-ATPase), thereby decreasing the cytoplasmic Na^+^ concentration [40]. The vacuolar Na^+^/H^+^ antiporter NHX1 helps compartmentalize Na^+^ in a process involving the proton gradient produced by the vacuolar membrane H^+^-ATPase (V-H^+^-ATPase) and H^+^-pyrophosphatases (H^+^-PPases), which limits the Na^+^ transported to the shoots and the toxic effects of Na^+^ in plants [41, 42]. In the current study, the 100 mM NaCl treatment resulted in a sharp increase in the Na^+^ content and a significant decrease in the K^+^ content in the *Csbpc2* mutants and WT control, leading to a significant increase in the Na^+^/K^+^ ratio. Accordingly, a high Na^+^ concentration impedes K^+^ absorption, which has detrimental effects on intracellular Na^+^ and K^+^ homeostasis. However, compared with the WT plants, the mutants accumulated more Na^+^ and less K^+^, resulting in a significantly higher Na^+^/K^+^ ratio (Fig. 5). In addition, on day 8 of the salt treatment, the root and leaf Na^+^ efflux rates were significantly lower for the *Csbpc2* mutants than for the WT control, whereas the opposite trend was observed for the K^+^ efflux rates (Fig. 6). Moreover, under saline conditions, the mutant plants had lower V-H^+^-ATPase and PM-H^+^-ATPase activities (Fig. 5). These findings imply that a non-functional CsBPC2 inhibits the salt-induced activation of V-H^+^-ATPase and PM-H^+^-ATPase. Therefore, under salt stress conditions, the proton motive force was weakened in the *Csbpc2* mutants, preventing them from excreting or compartmentalizing the excessive intracellular Na^+^, resulting in increased Na^+^ contents and decreased K^+^ contents as well as increased ion toxicity.

### CsBPC2 regulates ABA synthesis and ABA signal pathway under salt stress

Salinity stress can induce the rapid accumulation of ABA, which activates the signaling pathway that mediates stomatal closure and plant salt tolerance [43]. The ABA receptors PYR/PYL/RCARs, clade A PP2Cs, and SnRK2.2/3/6 constitute the core components of ABA signal transduction pathways, in which PP2Cs are the key negative regulators of early ABA signaling [44, 45]. Under saline conditions, PYR/PYL/RCARs bind to the accumulated ABA and then interact with PP2Cs (e.g. ABI1, ABI2, and AHG1) to prevent them from inhibiting SnRK2.2/3/6, which ultimately leads to the activation of ABA signal transduction pathways [5]. Transcription factors, such as AREB1, AREB2, and ABI5, phosphorylated by SnRK2s can activate the expression of stress-related genes downstream of the ABA signaling pathway to alleviate the damages caused by diverse stresses. Furthermore, SnRK2s can also phosphorylate SLAC1, which then mediates Cl^−^ efflux, while the phosphorylated KAT1 is inactive (i.e. no K^+^ influx), which leads to a decrease in intracellular ion contents, with the resulting decrease in cell turgor pressure causing stomata to close [5, 46, 47]. The ABA-induced stomatal closure restricts plant transpiration, which is crucial for decreasing the intake of salt by leaf tissues and enhancing plant tolerance to salt stress [48].

In our previous study, the application of ABA induced *CsBPC2* expression and inhibited the germination of seeds from tobacco plants overexpressing *CsBPC2*, implying CsBPC2 may mediate ABA signaling [22]. In this study, to further explore the regulatory effects of CsBPC2 on ABA biosynthesis and the ABA signaling pathway, the ABA contents and the transcription of ABA signaling-related genes were analysed. Interestingly, under normal growth conditions, the ABA contents were significantly higher in the *Csbpc2* mutants than in the WT plants (Fig. 7A). Similarly, the *CsABI1*, *CsABI5*, *CsAHG1*, *CsAREB1*, *CsAREB2*, *CsSLAC1*, *CsSnRK2.3*, *CsSnRK2.6.1*, and *CsSnRK2.6.2* expression levels were significantly higher in the mutants than in the WT control (Fig. 7), suggesting the high ABA contents promoted ABA signal transduction. Thus, a mutation to *CsBPC2* appears to enhance ABA signal transduction under normal growth conditions. Numerous investigations demonstrated that BPC TFs are involved in the regulation of a series of genes related to plant growth and development and that mutations to *BPC* genes cause both vegetative and reproductive defects [14, 22]. However, we did not detect any obvious vegetative or reproductive developmental defects in the *Csbpc2* mutants, possibly because the lack of a functional CsBPC2 suppressed several gene regulatory networks related to growth and development, while enhancing other signaling pathways. For example, the activation of the ABA signaling pathway may lead to increased ABA contents and ABA signal-related gene expression levels. However, this will need to be experimentally verified. Nevertheless, in the present study, the ABA content increased significantly in the WT control on day 8 of the salinity treatment and then decreased, but the ABA content in the mutant plants was relatively stable. In addition, the ABA content was significantly higher in the WT plants than in the mutants after 8 days of the salt treatment (Fig. 7). These results indicate CsBPC2 is participated in regulation of ABA synthesis under saline conditions, with mutations to *CsBPC2* resulting in inhibited ABA production. The *CsABI1*, *CsABI5*, *CsAHG1*, *CsAREB1*, *CsAREB2*, *CsSnRK2.6.1*, and *CsSnRK2.6.2* transcription levels were significantly lower in the mutants than in the WT plants following the salt treatment for 8 or 15 days (Fig. 7), implying that CsBPC2 is necessary for ABA-mediated signal transduction. Many studies revealed that BPC TFs control plant responses to hormones, including ethylene [14], cytokinins [17, 21], and brassinosteroids [11], but the roles of these TFs in response to ABA have not been thoroughly characterized. Collectively, the results of the current study suggest *CsBPC2* encodes an important TF that contributes to cucumber responses to abiotic stresses, including salinity, as well as ABA synthesis and signal transduction. Hence, the underlying regulatory mechanisms will need to be elucidated.

### Conclusion

**Figure 8:**
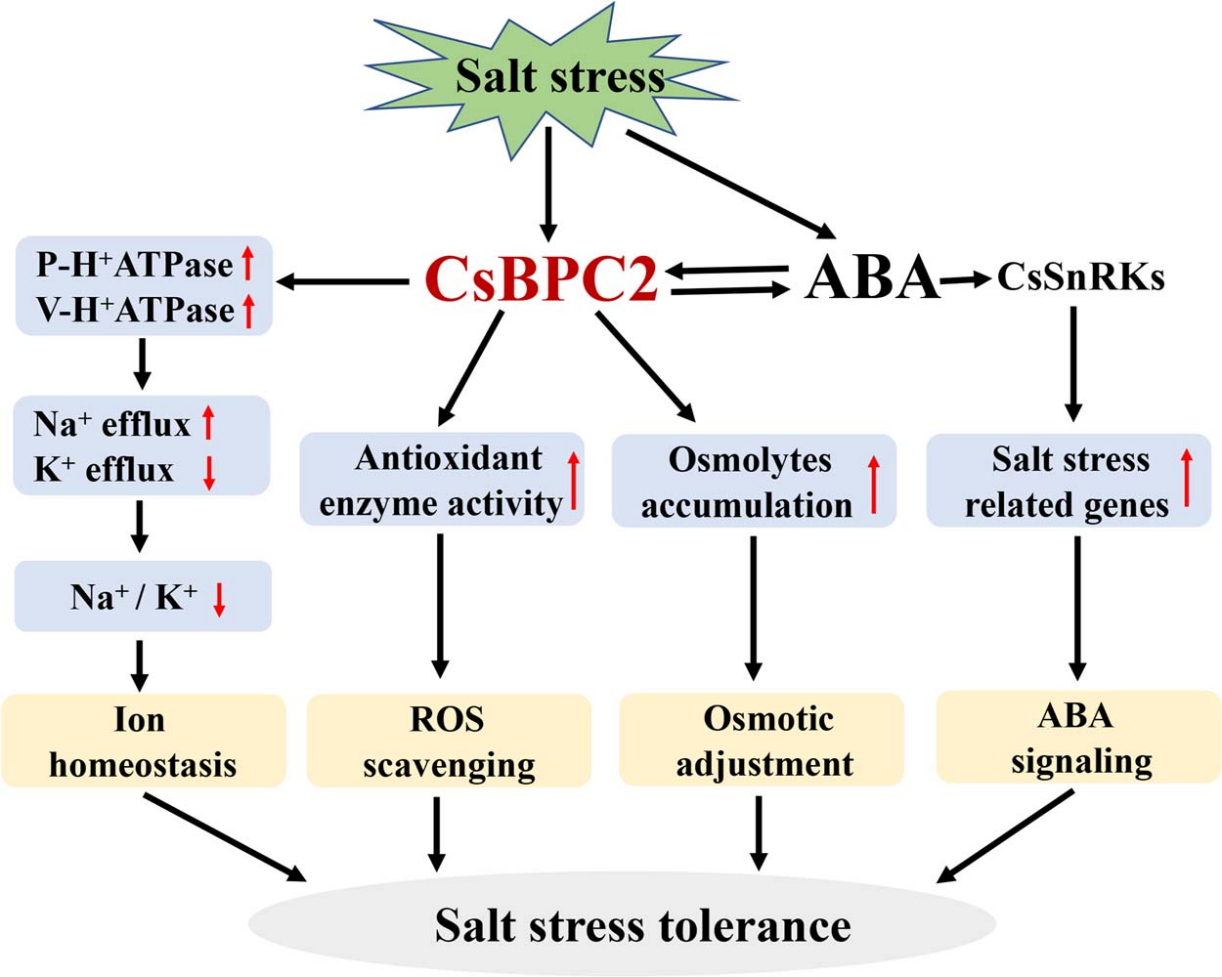
Schematic illustration of the CsBPC2 regulatory mechanism in response to salinity stress. Salinity stress induces ABA biosynthesis. The resulting ABA promotes the transcription of *CsBPC2*, which in turn regulates ABA biosynthesis and ABA signaling-related gene expression, thereby activating ABA signal transduction pathways. Additionally, salt stress induces the expression of *CsBPC2* directly, which increases the accumulation of osmolytes, antioxidant enzyme activities, and V-H^+^-ATPase and PM-H^+^-ATPase activities. These changes ultimately enhance plant salt stress tolerance through the associated osmotic adjustments, ROS scavenging, and ion homeostasis pathways.

In this study, we revealed the effects of the cucumber BPC2 TF on salt stress tolerance. We also developed a model for the regulatory effects of CsBPC2 (Fig. 8). Briefly, salt stress induces *CsBPC2* expression, and mutated *CsBPC2* leads to decreased osmolyte contents, antioxidant enzyme activities, and V-H^+^-ATPase and PM-H^+^-ATPase activities. That is, a mutated *CsBPC2* leads to inhibited osmotic adjustments and ROS scavenging, while also causing an ionic imbalance. These changes negatively regulate plant salt stress tolerance. Of course, overexpressed *CsBPC2* may show the opposite result. Furthermore, CsBPC2 is also involved in the ABA signaling pathway and is required for salt-induced ABA biosynthesis and the transcription of ABA signaling-related genes. There have recently been many breakthroughs in the research on the regulatory functions of BPCs on plant growth and development, but there remains an urgent need for studies on BPC functions associated with stress responses and ABA signal transduction. The data presented herein suggest CsBPC2 may be an important factor for cucumber responses to abiotic stresses, especially salt stress, and the ABA signaling pathway. However, the regulatory mechanisms modulating BPC-mediated stress responses should be explored in more detail and the stress-related target genes will need to be identified.

## Materials and methods

### Vector construction and cucumber transformation

The CRISPR/Cas9 binary vector pKSE402 (described by Zhonghua Zhang), which contains a 35S-GFP expression cassette and the *CsU6* (cucumber *U6* gene) promoter for sgRNA expression, was modified from pKSE401 [49]. To generate the CRISPR/Cas9 plasmid, one *CsBPC2* sgRNA sequence was designed using the Geneious software (Table S1, see online supplementary material). The sgRNA expression cassette was inserted into pKSE402 at the *BsaI* site as previously described [49]. The constructed binary vector pKSE402-*CsBPC2* was then inserted into EHA105 (*Agrobacterium tumefaciens* strain) cells for subsequent genetic transformation of cucumber cultivar ‘Changchunmici’ (maintained in our laboratory) as previously described [25], with some changes. To identify regenerated plants, GFP fluorescence was observed using the Leica MZ10F stereomicroscope (Leica Microsystems, Wetzlar, Germany). Genomic DNA was isolated from T_0_ lines with GFP fluorescence according to a CTAB-based method. The primers specific for sequences flanking the sgRNA were used for a PCR amplification (Table S2, see online supplementary material). The PCR products were then extracted and cloned into the pEASY-Blunt Simple Cloning Vector (TransGen Biotech, Beijing, China). To analyse the editing efficiency, approximately 10 clones from each GFP-positive plant were randomly selected and sequenced. The sequences were aligned using the Geneious software. The seeds derived from the T_0_ lines with a mutated gene were examined for GFP fluorescence. The GFP-negative seeds were subsequently sown. Genomic DNA was extracted from GFP-negative seedlings, after which the 486-bp sequence around the target site was amplified by PCR. The PCR products were then sequenced. The heterozygous GFP-negative T_1_ mutants were selfed and purified. Finally, the transgene-free *Csbpc2* homozygous mutants were obtained and verified by sequencing.

### Plant growth and salt treatments

The seeds of WT *Cucumis sativus* L. cv. ‘Changchunmici’ and homozygous *Csbpc2* mutants Line 1 (31 deleted nucleotides; Fig. 1L) and Line 2 (64 deleted nucleotides; Fig. 1L) were sown in plastic trays containing peat, vermiculite, and perlite (v/v/v = 2:1:1) in a climate chamber. When the seedlings reached one-leaf stage, five uniformly growing plants were transferred to 5 L plastic tanks (33 cm × 25 cm × 11 cm) containing Hoagland nutrient solution [22]. When the seedlings reached two-leaf stage, added 100 mM NaCl to the nutrient solution. Seedlings were cultivated under a 12 h day (25°C)/12 h night (18°C) photoperiod with 60%–80% relative humidity, 350 μmol·m^−2^·s^−1^ of light intensity. The nutrient solution was replaced every 2 days before and after the salt stress treatment. Samples were collected at 0, 3, and 8 days for the analysis of net Na^+^ and K^+^ flux, whereas the other indices were analysed using samples obtained at 0, 8, and 15 days.

### MDA content and electrolytic leakage assays

Electrolyte leakage (%) was determined as previously described [50]. Briefly, approximately 0.1 g fresh leaf disks were collected from all lines and soaked in 10 mL distilled deionized water in glass tubes. The samples were incubated at 32°C for 2 h and then the initial electrical conductivity (EC1) was measured using a conductivity meter FE30(Mettler Toledo,Switzerland). The samples were boiled for 20 min and cooled to room temperature before the final electrical conductivity (EC2) was measured. The electrical conductivity of deionized water (EC3) was also measured (i.e. background value). Electrolyte leakage was calculated using the following formula: EL (%) = (EC1 − EC3)/ (EC2 − EC3) × 100.

The MDA content was determined as described by Hu *et al.* [51], with some modifications. Briefly, 0.3 g fresh leaf samples were added to 3 mL phosphoric acid buffer (50 mM, pH 7.8) and ground. After centrifuging at 12000 rpm for 20 min at 4°C, 1 mL supernatants (1 mL deionized water as the control) were transferred to glass tubes. After adding 2 mL 0.67% thiobarbituric acid, the samples were boiled for 15 min, cooled to ambient temperature, and centrifuged at 12000 rpm for 20 min. The absorbance (at 600, 532, and 450 nm) was recorded.

### Proline and soluble sugar contents assays

The proline content was measured as previously described [52]. Leaf samples were dried and ground to a powder, after which 0.03 g ground samples were transferred to glass tubes. After adding 5 mL 3% (w/v) sulfosalicylic acid, the samples were boiled for 10 min, cooled to ambient temperature, and filtered. A 2-mL aliquot of each filtrate (2 mL deionized water as the control) was mixed with 2 mL acetic acid and 3 mL acid ninhydrin. The resulting solutions were boiled for 40 min. After cooling to room temperature, 5 mL toluene was added to the solutions, which were mixed thoroughly. Finally, the absorbance (at 520 nm) of the toluene phase was recorded.

The soluble sugar content was measured using a modified version of the method described by Fairbairn [53]. Leaf samples were dried and ground to a powder. For each sample, 0.03 g powdered material was added to 10 mL deionized water in a glass tube. The samples were boiled twice for 30 min each and then filtered. Deionized water was added to the filtrate for a final volume of 50 mL. A 1-mL aliquot of the diluted filtrate was mixed with 1 mL deionized water (2 mL deionized water as the control), 0.5 mL anthrone ethyl acetate, and 5 mL H_2_SO_4_ and then boiled for 1 min. After cooling to room temperature, the absorbance (at 630 nm) was recorded.

### Analyses of the H_2_O_2_ content, O_2_^.−^ production rate, and antioxidant enzyme activities

The H_2_O_2_ content was measured as a published TiCl_4_ precipitation method [54]. The O_2_^.−^ production rate was calculated according to Tian *et al.* [55]. The antioxidant enzyme SOD activity was determined using nitroblue tetrazolium according to Prochazkova *et al.* [56], whereas the POD and CAT activities were measured as described by Cakmak and Marschner [57]. The APX and DHAR activities were analyzed as described by Nakano and Asada [58].

### Determination of Na^+^ and K^+^ contents

Cucumber leaves and roots were detached and washed with deionized water. They were dried at 105°C for 15 min and then at 70°C to a constant weight. The oven-dried samples were ground to a fine powder. For each sample, 5 mg ground material was added to a digester tube containing 5 mL concentrated HNO_3_. The samples were digested in a microwave digestion apparatus (Milestone, Italy) until they were clear. After boiling the samples, they were diluted to 25 mL with deionized water and thoroughly mixed. Finally, the Na^+^ and K^+^ contents were determined using the 5300 DV inductively coupled plasma emission spectrometer (PerkinElmer, USA).

### PM-H^+^-ATPase and V-H^+^-ATPase activity assays

Cucumber leaves (approximately 0.1 g per sample) were ground to a powder using a refrigerated grinding machine. Next, 0.9 mL (weight:volume = 1:9) phosphate buffer (0.05 M, pH 7.4) was added and the solution was mixed before being centrifuged at 4000 rpm for 20 min at 4°C. The supernatants were used for the PM-H^+^-ATPase and V-H^+^-ATPase activity assays, which were completed using a commercial enzyme-linked immunosorbent assay kit (Jiangsu Meimian Industrial Co., Ltd., China).

### Determination of Na^+^ and K^+^ fluxes

After cucumber seedlings were cultured in NaCl for 3 and 8 days, the roots and leaves were collected for the analysis of the net flux of Na^+^ and K^+^ using a commercial NMT system (youngerusa.com; xuyue.net). First, the roots were fixed to the bottom of a Petri dish and then immersed in the measuring solution (0.1 mM CaCl_2_, 0.1 mM KCl, and 0.5 mM NaCl, pH 5.8) for 20 min prior to the ion flux measurement. A flux microsensor was used to analyse the net flux of Na^+^ and K^+^ in the root meristematic zone (500 and 600 μm from the root apex). Each site was examined for 5 min. All experiments were completed using eight biological replicates.

Cucumber leaves were detached and then the lower epidermis was removed using a tweezer. The leaf samples were then fixed to the bottom of a Petri dish and immersed in the measuring solution (0.1 mM CaCl_2_, 0.1 mM KCl, and 0.5 mM NaCl, pH 5.8) for 6 h prior to the ion flux measurement. The mesophyll tissue was located using a microscope and then the flux microsensor was placed approximately 50 μm from the leaf surface. Two sites were examined for 5 min each. All experiments were performed using eight biological replicates.

The ion flux data were recorded using the imFluxes (version 2.0) software (xuyue.net), with positive value representing ion efflux, and negative value representing ion influx.

### Determination of ABA content

Cucumber leaves (approximately 0.5 g per sample) were collected after 0, 8, and 15 d of the salt treatment and stored at −80°C*.* The ABA content was determined by Wuhan Metware Biotechnology Co., Ltd (Wuhan, China) using an LC-ESI-MS/MS system (HPLC, Shim-pack UFLC SHIMADZU CBM30A system, www.shimadzu.com.cn/; MS, Applied Biosystems 6500 Triple Quadrupole, www.appliedbiosystems.com.cn/).

### QRT-PCR analysis

The RNA of cucumber leaves was isolated following the instructions of the RNA prep pure Plant Kit (TANGEN) and first-strand cDNA was synthesized using PrimeScript™ RT reagent Kit with gDNA Eraser (TaKaRa). Quantitative RT-PCR of 10 ABA signaling related genes were performed according to instructions of the SYBR® Premix Ex Taq™ Kit (TaKaRa) using a Mx3000P real-time PCR instrument (Agilent). Each sample was performed in triplicate. Relative gene expression was analysed according to the 2^−△△Ct^ method [59]. Primers for the quantitative realtime PCR are listed in Table S3 (see online supplementary material). Gene screening method: All homologous protein sequences of the targer gene were obtained from TAIR (http://www.arabidopsis.org), and then used the obtained sequences to perform BLASTP in the Cucumber Genome Database (http://cucurbitgenomics.org/organism/2) to search for all homologous genes in cucumber. Finally, a phylogenetic tree was constructed with all downloaded protein sequences from *Arabidopsis* and Cucumber Genome Database, and cucumber gene with the highest homology to the target gene in *Arabidopsis* was identified as the candidate gene.

### Statistical analysis

Statistical analyses were performed by an analysis of variance (ANOVA) using SAS 9.2 software (SAS Institute, Cary, NC, USA).

## Acknowledgments

This work was supported by the National Natural Science Foundation of China (No.31972480 and 32260800), the Earmarked fund for Modern Agro-industry Technology Research System in China (CARS-25-C-01), the Science and Technology Innovation Program of the Chinese Academy of Agricultural Sciences (CAASASTIP-IVFCAAS), the Key Laboratory of Horticultural Crop Biology and Germplasm Innovation, Ministry of Agriculture, China, and the Natural Science Foundation of Jiangxi Province (20212BAB215004).

## Author contributions

Y.L. and X.Y. conceived and designed the research. S.L., L.M., Q.D., L.L. and A.S. performed the experiments and analysed the data. Y.Y., C.H. and J.W. made important comments on design of the research and article writing. S.L. wrote the paper. Y.L. and M.S. revised the paper. All authors read and approved the final version of the paper.

## Data availability

All data in this study are available from the corresponding author on reasonable request.

## Conflict of interest

The authors declare no competing interests.

## Supplementary data

Supplementary data is available at *Horticulture Research* online.

## Supplementary Material

Web_Material_uhad051
