# Supplementary material for: Multifaceted regulatory functions of CsBPC2 in cucumber under salt stress conditions
Source: Hortic Res. 2023 Mar 15;10(5):uhad051. doi: 10.1093/hr/uhad051 (PMC10194891; doi:10.1093/hr/uhad051)
Supplement: Web_Material_uhad051 [file web_material_uhad051.zip › Supplemental Data.docx]

**Supplemental Data**

Supplemental Data S1. Nucleic acid and protein sequences of CsBPC2 gene in wild-type and *Csbpc2* mutant plants.

**A: Nucleic acid sequence of CsBPC2 gene**

**Nucleic acid sequence of CsBPC2 gene in wild-type plants**

ATGGATGGTGATGCTTTGAACATGCGTAATTGGGGTTACTATGAACCATCTTTGAAAGCGCATCTTGGACTGCAGCTCATGTCCACAATTGGTGAGCGAGATGTGAAACATTTTATGCCTGGACGGGACCCGTCAGCTATTGTTAACATGAATGCCGCATTTCATCCACGGGAGTCTGTTGTTTCTGAAGCACCGGTAGCGACGAACTGGGCGAGGGATGGTTGGATAAATCATAGGGACAAGCTTTTCAATGTGTTATCCCCCAATACTAGTTATTCTCTGCTTGCAGAGACGTCAGCGGCACAACCTTTGCAAATTTTACAGCCACTTGACACATCAAGAGATGAAATGGTTCTCAAGATTGAAGAACCACCTGTGAAGAAGGGAACTAAACAACCAAAGAAACGACAGAATGGAGGTGCTCCCAAAACTCCGAAACCAAAGAAGCCTCGAAAGCCTAAAAATAATGATCCTTCGGTTCAACAGGTGAAGGCACCAAAAAAGAAGATGGAGCTTGTTATAAATGGGTTTGACATGGATATATCTAGTATCCCAATTCCAGTATGTTCTTGCACTGGAACTCCTCACCAATGTTATAGATGGGGCTATGGTGGCTGGCAATCAGCTTGTTGTACCACAAGTTTATCTCTACATCCTTTGCCAATGAGTGAGAAGCGGCGAGGTGCAAGAATTGCTGGTCGAAAAATGAGTCAAGGTGCTTTTAAGAAGGTTTTGGAGAAACTAGCAGCTCAAGGCTATAACTTTTCTAACCCAATTGATTTAAGAAGCCATTGGGCAAGGCATGGGACCAATAAGTTTGTCACAATCAGGTGTACTAATCCTGTCTCAAATTAA

**Nucleic acid sequence of CsBPC2 gene in mutant plant line1**

ATGGATGGTGATGCTTTGAACATGCGTAATTGGGGTTACTATGAACCATCTTTGAAAGCGCATCTTGGACTGCAGCTCATGTCCACAATTGGTGAGCGAGATGTGAAACATTTTATGCCTGGACGGGACCCGTCAGCTATTGTTAACATGAATGCCGCATTTCATCCACGGGAGTCTGGGCGAGGGATGGTTGGATAAATCATAGGGACAAGCTTTTCAATGTGTTATCCCCCAATACTAGTTATTCTCTGCTTGCAGAGACGTCAGCGGCACAACCTTTGCAAATTTTACAGCCACTTGACACATCAAGAGATGAAATGGTTCTCAAGATTGAAGAACCACCTGTGAAGAAGGGAACTAAACAACCAAAGAAACGACAGAATGGAGGTGCTCCCAAAACTCCGAAACCAAAGAAGCCTCGAAAGCCTAAAAATAATGATCCTTCGGTTCAACAGGTGAAGGCACCAAAAAAGAAGATGGAGCTTGTTATAAATGGGTTTGACATGGATATATCTAGTATCCCAATTCCAGTATGTTCTTGCACTGGAACTCCTCACCAATGTTATAGATGGGGCTATGGTGGCTGGCAATCAGCTTGTTGTACCACAAGTTTATCTCTACATCCTTTGCCAATGAGTGAGAAGCGGCGAGGTGCAAGAATTGCTGGTCGAAAAATGAGTCAAGGTGCTTTTAAGAAGGTTTTGGAGAAACTAGCAGCTCAAGGCTATAACTTTTCTAACCCAATTGATTTAAGAAGCCATTGGGCAAGGCATGGGACCAATAAGTTTGTCACAATCAGGTGTACTAATCCTGTCTCAAATTAA

**Nucleic acid sequence of CsBPC2 gene in mutant plant line2**

ATGGATGGTGATGCTTTGAACATGCGTAATTGGGGTTACTATGAACCATCTTTGAAAGCGCATCTTGGACTGCAGCTCATGTCCACAATTGGTGAGCGAGATGTGAAACATTTTATGCCTGGACGGGACCCGTCAGCTATTGTTAACATGAATGCCGCATTTCATCCACGGGAGTCTGTTGTTTCAATGTGTTATCCCCCAATACTAGTTATTCTCTGCTTGCAGAGACGTCAGCGGCACAACCTTTGCAAATTTTACAGCCACTTGACACATCAAGAGATGAAATGGTTCTCAAGATTGAAGAACCACCTGTGAAGAAGGGAACTAAACAACCAAAGAAACGACAGAATGGAGGTGCTCCCAAAACTCCGAAACCAAAGAAGCCTCGAAAGCCTAAAAATAATGATCCTTCGGTTCAACAGGTGAAGGCACCAAAAAAGAAGATGGAGCTTGTTATAAATGGGTTTGACATGGATATATCTAGTATCCCAATTCCAGTATGTTCTTGCACTGGAACTCCTCACCAATGTTATAGATGGGGCTATGGTGGCTGGCAATCAGCTTGTTGTACCACAAGTTTATCTCTACATCCTTTGCCAATGAGTGAGAAGCGGCGAGGTGCAAGAATTGCTGGTCGAAAAATGAGTCAAGGTGCTTTTAAGAAGGTTTTGGAGAAACTAGCAGCTCAAGGCTATAACTTTTCTAACCCAATTGATTTAAGAAGCCATTGGGCAAGGCATGGGACCAATAAGTTTGTCACAATCAGGTGTACTAATCCTGTCTCAAATTAA

**B:The protein sequence of CsBPC2 gene**

**Protein sequence of CsBPC2 gene in wild-type plants**

MDGDALNMRNWGYYEPSLKAHLGLQLMSTIGERDVKHFMPGRDPSAIVNMNAAFHPRESVVSEAPVATNWARDGWINHRDKLFNVLSPNTSYSLLAETSAAQPLQILQPLDTSRDEMVLKIEEPPVKKGTKQPKKRQNGGAPKTPKPKKPRKPKNNDPSVQQVKAPKKKMELVINGFDMDISSIPIPVCSCTGTPHQCYRWGYGGWQSACCTTSLSLHPLPMSEKRRGARIAGRKMSQGAFKKVLEKLAAQGYNFSNPIDLRSHWARHGTNKFVTIRCTNPVSN*

**Schematic of the protein sequence**

**
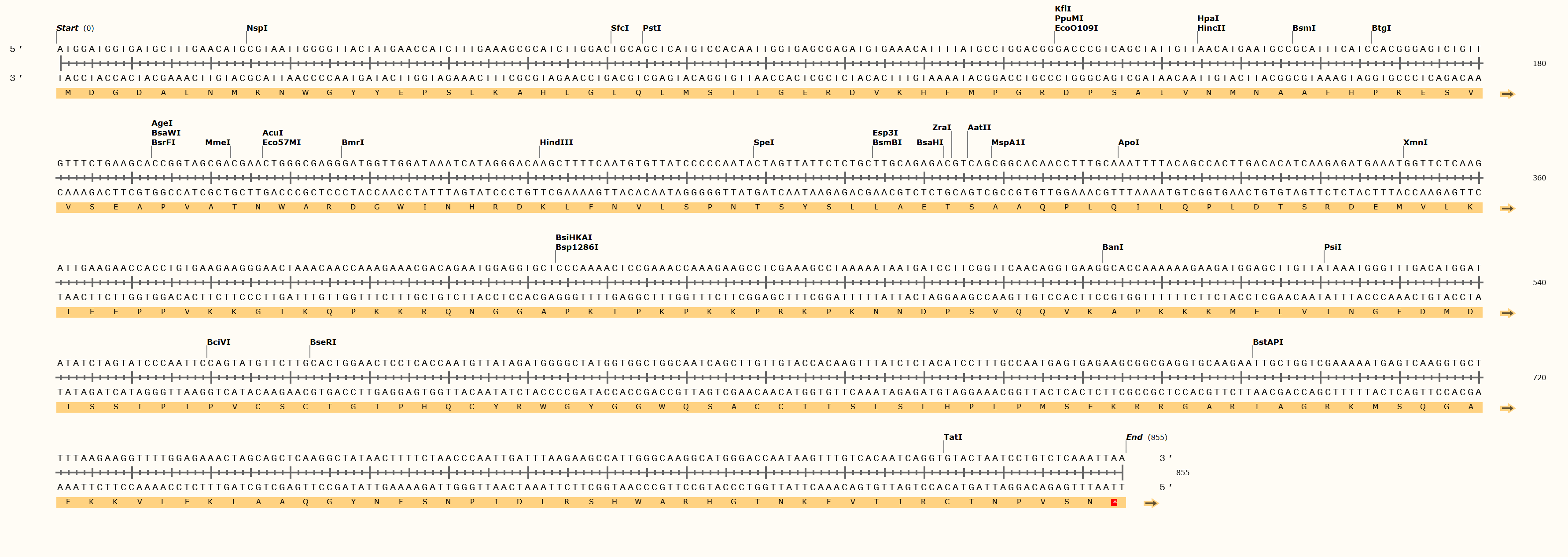
**

**Protein sequence of CsBPC2 gene in mutant plant line1**

MVLKIEEPPVKKGTKQPKKRQNGGAPKTPKPKKPRKPKNNDPSVQQVKAPKKKMELVINGFDMDISSIPIPVCSCTGTPHQCYRWGYGGWQSACCTTSLSLHPLPMSEKRRGARIAGRKMSQGAFKKVLEKLAAQGYNFSNPIDLRSHWARHGTNKFVTIRCTNPVSN*

**Schematic of the protein sequence**

**
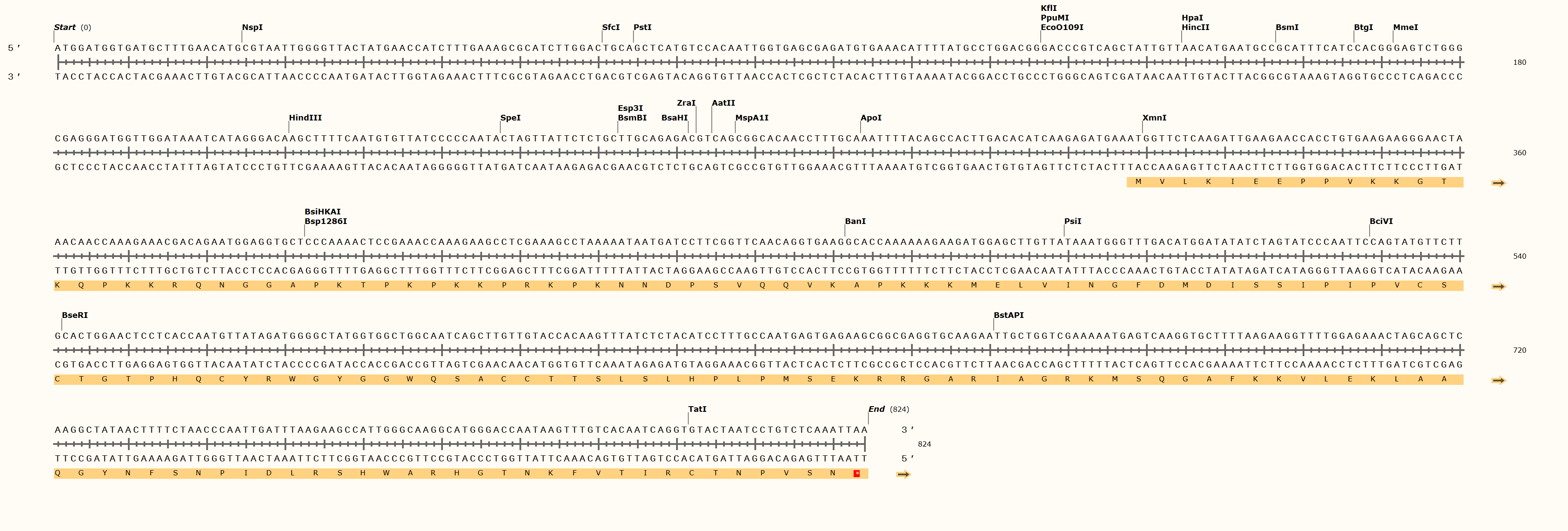
**

**Protein sequence of CsBPC2 gene in mutant plant line2**

**ORF1**

MDGDALNMRNWGYYEPSLKAHLGLQLMSTIGERDVKHFMPGRDPSAIVNMNAAFHPRESVVSMCYPPILVILCLQRRQRHNLCKFYSHLTHQEMKWFSRLKNHL*

**ORF2**

MVLKIEEPPVKKGTKQPKKRQNGGAPKTPKPKKPRKPKNNDPSVQQVKAPKKKMELVINGFDMDISSIPIPVCSCTGTPHQCYRWGYGGWQSACCTTSLSLHPLPMSEKRRGARIAGRKMSQGAFKKVLEKLAAQGYNFSNPIDLRSHWARHGTNKFVTIRCTNPVSN*

**Schematic of the protein sequence**


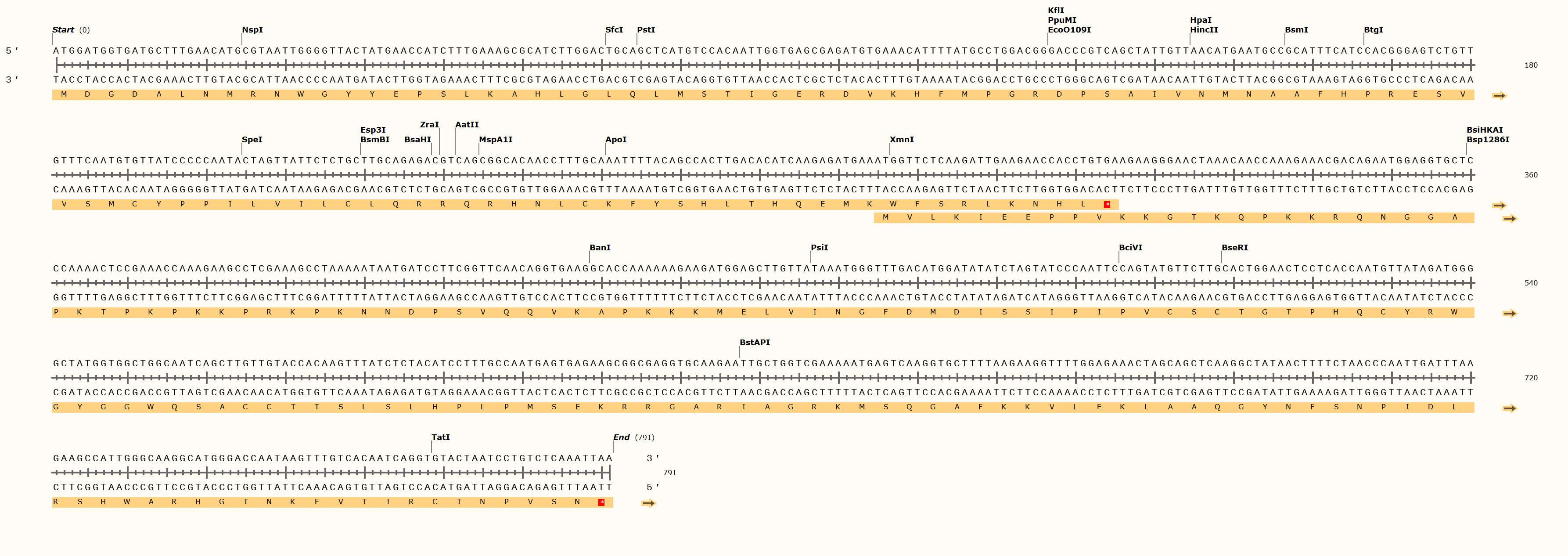


Supplemental Table S1. Primers used for vector construction

| **Primer name** | **Sequence** |
| --- | --- |
| CsBPC2-sgRNA | 5’- TCTGTTGTTTCTGAAGCAC CGG -3’ |
| CsBPC2-sgRNA-F | 5’- ATTGTCTGTTGTTTCTGAAGCAC -3’ |
| CsBPC2-sgRNA-R | 5’- AAACGTGCTTCAGAAACAACAGA -3’ |

Supplemental Table S2. Primers used for genetic transformation

| **Primer name** | **Sequence** |
| --- | --- |
| CsBPC2-target-test-F | 5’- GGATGGTGATGCTTTGAAC -3’ |
| CsBPC2-target-test-R | 5’- ACCTGTTGAACCGAAGGA -3’ |
| GFP-F | 5’- ATGGTGAGCAAGGGCGAG -3’ |
| GFP-R | 5’- TTAAGATCTGTACAGCTCGTCCATGC -3’ |

Supplemental Table S3. Primers used for RT-qPCR analysis

| **Gene ID** | **Gene** | **Forward primer (5’-3’)** | **Reverse primer (5’-3’)** |
| --- | --- | --- | --- |
| Csa2G357210.1 | CsAREB1 | ATGCCAATGGAGTTCGAGCA | CCATTCTTCCACCCTGCAGT |
| Csa7G073570.1 | CsAREB2 | ATGGTGGGAACTTGCAGAGG | GCTGGCATTGGTTCGATTCC |
| Csa3G776860.1 | CsABI5 | GCTTACACTTCCTGCACCCT | CCGGATTCTGAGAGTTGGCA |
| Csa3G038090.1 | CsSLAC1 | GCTTACACTTCCTGCACCCT | CCGGATTCTGAGAGTTGGCA |
| Csa1G448920.1 | CsKAT1 | ATCTTCTCGTCGACCACCCT | AGCCTTGCGAAAAGAGAGCT |
| Csa7G074880.1 | CsSnRK2.6.1 | GCCGCCATCTGATCTCTAGG | GGGCTCTTCGTATTGGCTGT |
| Csa2G236620.1 | CsSnRK2.6.2 | CAAAGAACCTACCGACCGAATTG | TCCAAACACCCTGTCAGATACTG |
| Csa2G286490.1 | CsSnRK2.3 | TCTTGACCACTACCCATCTTGC | TGAAAGAAGAAGCGAGCCTCAT |
| Csa3G604110.1 | CsABI1 | CGCTTGCATCGGATTGTGG | GGCAACGCAATTCCATCCC |
| Csa3G178550.1  Csa6G484600 | CsAHG1  CsActin | TCTACGATGGACATGGAGGC  TTCTGGTGATGGTGTGAGTC | CATTCTCTCAAAGCTCCGCC  GGCAGTGGTTGTGAACATG |
